# Editorial: Revisiting the challenges and opportunities in cancer drug resistance

**DOI:** 10.3389/fmolb.2024.1497754

**Published:** 2024-09-30

**Authors:** Tikam Chand Dakal, Narendra Kumar Sharma, Amit Sharma

**Affiliations:** ^1^ Genome and Computational Biology Lab, Department of Biotechnology, Mohanlal Sukhadia University, Udaipur, Rajasthan, India; ^2^ Department of Bioscience and Biotechnology, Banasthali Vidyapith, Tonk, Rajasthan, India; ^3^ Center for Integrated Oncology (CIO), Department of Integrated Oncology, University Hospital Bonn, Bonn, Germany

**Keywords:** cancer, drug resistance, challenges, precision medicine, treatment

Cancer drug resistance remains a significant challenge in oncology, hindering the effectiveness of chemotherapeutic agents and leading to poor patient outcomes (Bukowski et al., 2020). Acquired drug resistance is also a prevalent cause of the unfavorable prognosis and survival results in cancer. Disease recurrence can also be attributed to drug resistance. Drug resistance is influenced by several causes, including genetic and epigenetic changes, modifications in drug targets, inhibition of apoptosis, increased drug efflux, and distinct cellular and molecular pathways. Drug resistance in cancer cells can arise through various mechanisms, including modulation of the tumor microenvironment, alteration of drug targets, and rewiring of genetic, epigenetic, and metabolic processes. Cancer cells within a tumor exhibit significant heterogeneity, with diverse subpopulations, such as cancer stem cells, displaying distinct drug response patterns. The heterogeneity of cancer cells plays a critical role in drug resistance by transforming the tumor microenvironment from an anti-tumorigenic to a pro-tumorigenic state (Sun and Yu, 2015). Recent findings establish the crucial significance of tumor microenvironment and cancer stem cells in the development of drug resistance, which is the primary cause of cancer recurrence and its inability to be cured. The presence of genetic Research Topic across individuals and tumors, together with the ability of cancer to evade conventional treatments, further complicates the management of medication resistance. Despite rapid development in the design of novel chemotherapy drugs, there is still a lack of effective agents targeting the advanced stages of cancer, namely invasion and metastasis.

Through this Research Topic, we intended to illuminate promising lines of inquiry, namely focusing on new approaches recently discovered in the field, including fundamental genetics, genomic engineering, omics techniques, artificial intelligence, and machine learning. Our goal was to uncover the molecular basis of drug resistance in various cancers, so guiding clinicians in designing and developing therapeutic strategies to improve therapeutic drug sensitivity and overcome resistance. Research presented in this topic showcase novel approaches and openly address the limitations of existing therapeutic remedies. Research papers collected for this topic elucidate the key mechanisms underlying cancer drug resistance and explore potential therapeutic strategies to overcome this obstacle.

Several mechanisms have been identified that confer drug resistance in cancer cells. These include increased drug efflux, alterations in drug targets, activation of alternative signaling pathways, and enhanced DNA repair mechanisms (Fernández-Lázaro, 2018). Cancer stem cells have emerged as key contributors to the development of drug resistance and tumor relapse. These cells possess remarkable abilities to resist conventional treatments, driving disease recurrence and metastasis. The unique properties of cancer stem cells, such as self-renewal, differentiation potential, upregulated surface membrane immune inhibitory ligands, and the release of various chemo/cytokines, collectively contribute to therapy resistance and pose significant challenges in achieving long-term remission. The tumor microenvironment also plays a crucial role in mediating drug resistance, with cancer stem cells, stromal cells and immune cells being reprogrammed by cancer cells to secrete factors that support tumor progression and suppress cell death programming (apoptosis). Understanding the stepwise drug-resistant mechanisms gained by different cancer cells is crucial for the development of effective therapeutic strategies. Targeting cancer stem cells and the tumor microenvironment may open up new avenues for novel combination therapies that can help overcome drug resistance and potentially improve patient outcomes (Jin and Jeong, 2023; Fernández-Lázaro, 2018; Bukowski et al., 2020; Sun and Yu, 2015).

The integration of multi-omics technology, computational methodologies, and the creation of tailored medicines presents novel approaches to address the issue of drug resistance. In conclusion, the challenge of multidrug resistance in cancer remains a significant obstacle in achieving effective and durable cancer therapy. Understanding the complex and heterogeneous mechanisms underlying drug resistance is crucial for developing novel therapeutic strategies. Advancements in cancer immunotherapy, the creation of immune checkpoint inhibitors or targeted small molecules, and the repurposing of non-oncology medications have expanded the scope of ongoing pharmaceutical research. The advancement of nanotechnology-based drug delivery systems has provided promising avenues to overcome these resistance mechanisms and improve patient outcomes. In this context, the application of emerging technologies, such as CRISPR-Cas9-based gene editing, may prove valuable in combating multidrug resistance genes and enhancing the efficacy of cancer treatments.
